# Distinguish microphase-separated structures of diblock copolymers using local order parameters

**DOI:** 10.1038/s41598-024-74525-y

**Published:** 2024-10-13

**Authors:** Fumiki Takano, Masaki Hiratsuka, Kazuaki Z. Takahashi

**Affiliations:** 1 Kogakuin University, 1-24-2 Nishi-Shinjuku, Tokyo, 163-8677 Japan; 2 National Institute of Advanced Industrial Science and Technology (AIST), Research Center for Computational Design of Advanced Functional Materials, Central 2, 1-1-1 Umezono, Tsukuba, Ibaraki 305-8568 Japan

**Keywords:** Chemical physics, Thermodynamics, Computational science, Computer science, Polymers

## Abstract

The microphase-separated structures of block copolymers are inherently highly ordered local structures, commonly characterized by differences in domain width and curvature. By focusing on diblock copolymers, we propose local order parameters (LOPs) that accurately distinguish between adjacent microphase-separated structures on the phase diagram. We used the Molecular Assembly structure Learning package for Identifying Order parameters (MALIO) to evaluate the structure classification performance of 186 candidate LOPs. MALIO calculates the numerical values of all candidate LOPs for the input microphase-separated structures to create a dataset, and then performs supervised machine learning to select the best LOPs quickly and systematically. We evaluated the robustness of the selected LOPs in terms of classification accuracy against variations in miscibility and fraction of block. The minimum local area size required for LOPs to achieve their classification performances is closely related to the characteristic sizes of the microphase-separated structures. The proposed LOPs are potentially applicable over a large area on the phase diagram.

## Introduction

Block copolymers are commonly used in automotive parts, electronic devices, and filtration membranes. Such polymers are composed of several blocks that spontaneously form microphase-separated structures, depending on the miscibility and fraction of these blocks^1,2^. For example, at high temperatures, block copolymers form homogeneous and disordered polymer melts. As the polymer melts are cooled, the miscibility decreases and the blocks form morphologies that are separated from each other in a manner that minimizes the contact between the blocks. Spontaneous formation of this morphology, known as self-assembly, is expected to play an important role in improving the functionality and manufacturing efficiency of nanodevices. In previous studies, the nanostructure of polymer thin films coated on semiconductor substrates was controlled to less than $10\,\text{nm}$^3–5^. Other studies have been conducted to reduce the time required for film formation in semiconductor manufacturing^6,7^. The development of porous polymer films based on co-continuous structures has also been actively investigated^8,9^. In developing these polymer products based on self-assembly, progress has been made in understanding the morphology, formation rate, and properties of the polymers.

The thermal and mechanical properties of block copolymer products are highly dependent on microphase-separated structures^10^. Transmission electron microscopy(TEM)^11–14^, small-angle X-ray scattering(SAXS)^11–13,15,16^, and neutron scattering(NS)^15,17^ are often used to identify these structures at the nanoscale. TEM images allow direct observation of nanoscale structures, but pre-processing of the sample may limit the structural information away from its inherent state. While scattering profiles have quantitative information about the shape and size of the structure, it is difficult to obtain even the molecular arrangement in real space. Therefore, Matsen et al. numerically calculated the segment profiles of polymer in lattice space based on self-consistent field theory and estimated equilibrium structures such as lamellar, gyroid, and cylindrical phases^18–21^. This is a numerical simulation method for analyzing the conformation of polymer chains and the curvature of domain surfaces, by numerically calculating the free energy described by the Flory–Huggins parameter $\chi$ and the fraction *f* of the polymer segments. Calculating the free energy of polymers in lattice space enables estimation of the phase equilibrium structures on a continuum-scale in accordance with *f* and $\chi$. However, averaged many-body interactions and fixed lattices of polymers inhibit understanding of the morphology formation caused by changes in external field and thermodynamic conditions^2,22,23^.

Molecular-level modeling solves the above problem, but there should not be a gap between the model and continuum-scale structural information. Therefore, backmapping, which estimates molecular-level structures based on continuum-scale structural information, has been considered^24–27^. Aoyagi et al. backmapped the microphase-separated structures of block copolymers from a continuum model to a coarse-grained molecular configuration^24^ by a density biased Monte Carlo (DBMC) method^28^. Pezeshkian et al. proposed a method to backmap the coarse-grained molecular configurations of lipid membranes from the continuum model of lipid membranes represented by triangular polygonal meshes^27^. Backmapping reveals molecular behaviors that cannot be easily discussed at the continuum-scale. For example, a continuum spherical structure was backmapped to a Kremer–Grest bead spring (KGBS) model^29,30^, in which polymer segments and their bonds are represented by beads and springs, and the entanglement and mechanical properties of the polymer chains during extension were evaluated^24,25^. Nevertheless, the relationship between polymer chain dynamics and the formation process of microphase-separated structures is still not fully understood. This is because microphase-separated structures are inherently highly ordered structures. Furthermore, there are limitations in characterizing the ordered structure simply by the width and curvature of the domains formed by polymer segments^31–33^.

Recently, attempts have been made to introduce local order parameters (LOPs) as a method of characterizing highly ordered molecular local structures. Various LOPs that have been independently devised over recent decades, with the help of machine learning, are useful in classifying various patterns of ordered structures beyond what was previously assumed. LOPs can accurately distinguish not only basic crystal structures such as face-centered cubic lattice, body-centered cubic lattice, and hexagonal close-packed^34^; but also ice polymorphs^35–37^, ordered structures in liquid crystals^38,39^, Weaire–Phelan structures^40^, and polymer lamellar structures^41^. Furthermore, some of these LOPs also explain global ordering by rationally describing the nucleation-to-percolation transition^38,39^. Such features of LOPs might provide new insights into the relationship between microphase-separated structures of block copolymers and polymer dynamics. In fact, it has been proposed to introduce the LOP into the Ginzburg–Landau-type free energy to describe the liquid–liquid phase separation structure and dynamics of water^42^, which is much simpler than that of block copolymers. The introduction of the LOP and Ginzburg–Landau equation also for block copolymers may provide a detailed description of the changes in mesoscale ordered structures associated with phase transitions. Furthermore, since the LOP is representative of molecular-scale ordered structures in this approach, it may lead to an understanding of the formation process of multiscale ordered structures. However, LOPs have not been evaluated in the structures of basic diblock copolymers.

In this study, we quickly and systematically searched for LOPs that can distinguish microphase-separated structures by machine learning from a large number of candidate LOPs. In particular, we focused on classifying four types of ordered structures among the microphase-separated structures that manifest in diblock copolymers. First, we calculated segment profiles on the lattice space corresponding to the lamellar, gyroid, and cylindrical structures based on self-consistent field theory, and then created each structure represented by the KGBS model by backmapping. Subsequently, we searched for LOPs that distinguish neighboring microphase-separated structures (disorder and cylinder, cylinder and gyroid, gyroid and lamella, as well as cylinder and lamella) on the phase diagram by using the Molecular Assembly structure Learning package for Identifying Order parameters (MALIO)^39^. MALIO computed the numerical values of all candidate LOPs for the input microphase-separated structures to create a dataset, and then performed supervised machine learning to select the best LOPs. Finally, we evaluated the applicability of the discovered LOPs over a large area on the phase diagram.

## Results and discussion

**Fig. 1:**
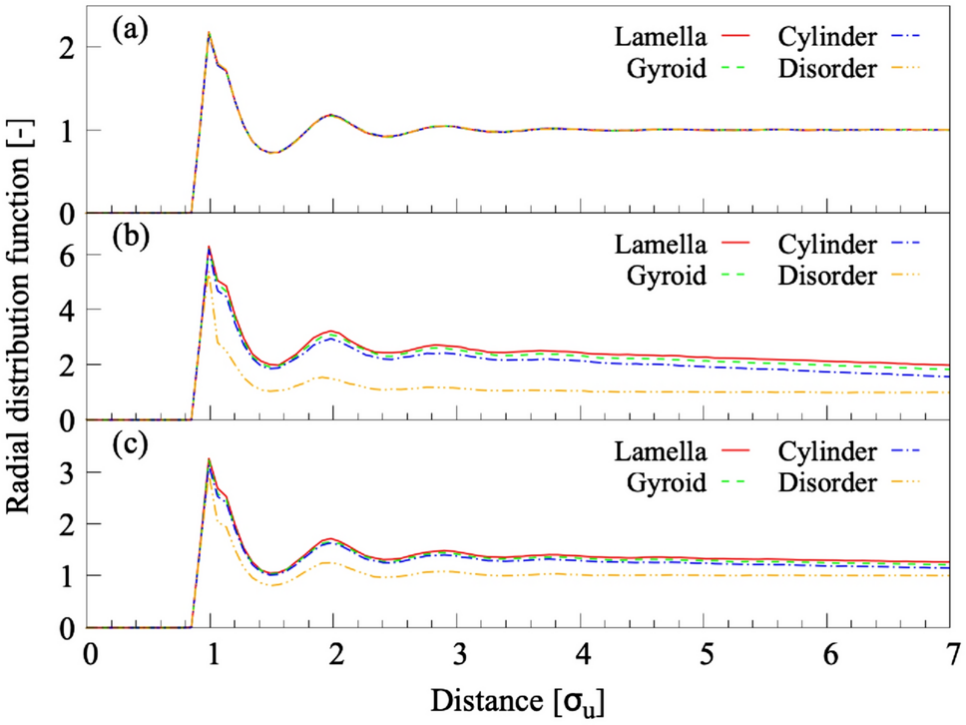
RDFs of microphase-separated structures. We calculated RDFs from (**a**) molecular structures with no distinction between the A- and B-blocks, (**b**) molecular structures consisting only of the A-blocks, and (**c**) molecular structures consisting only of the B-blocks. The solid red and dotted green, dotted blue, and dotted orange lines indicate lamellar, gyroid, cylindrical, and disordered structures, respectively. From (**b,c**), it is clear that it is easy to distinguish the disordered structure from ordered structures, but it is not easy to distinguish between ordered structures .

Before attempting to find LOPs that distinguish microphase-separated structures, we reveal the difficulty of characterizing these structures without LOPs by comparing the radial distribution functions (RDFs) for each structure on $f=0.33$. Figure 1 shows (a) RDFs computed without distinguishing between block types, (b) RDFs computed for only the A-blocks, and (c) RDFs computed for only the B-blocks. When block species were not distinguished, lamellar, gyroid, cylinder and disorder were barely distinguishable. In addition, even when we calculated RDFs for only the A- or B-blocks, the RDFs of the three ordered structures almost overlapped. In other words, the RDFs were only capable of distinguishing disordered structure from the rest. We evaluated LOPs with the aim of overcoming these difficulties and clearly distinguishing complex ordered structures with simple scalar parameters.

The search for LOPs that distinguish between disordered and cylindrical structures is expected to be the easiest problem in this study (see Fig. 1b,c), and therefore we considered two options in the LOP search through this problem. The first is whether or not to include block type (A or B) information in the structures to be input into MALIO. If the block species information is not given, it is unlikely that a useful LOP will be found because both structures are indistinguishable. In fact, the best-performing LOP proposed by MALIO had a classification accuracy of ca. 0.555, which implies a classification failure. Note that the classification accuracy defined in the Methods section is the correct response rate to multiple choice questions. The theoretical minimum correct response rate for classifying a large number of local structures into two names is 0.5, which is equal to the theoretical correct response rate for randomly answering a large number of multiple choice questions with two options. Given information on block species, it is efficient to find useful LOPs from only the information on the species (A) with the lowest number of particles in the structure. As described in the next section, “Disordered and cylindrical structures,” by inputting only the A-block structures into MALIO, we found LOPs with high classification accuracy.

The second is the choice of method for extracting local structure. MALIO extracts the local structure of all beads in the input structures by cutoff or count-up methods, but the difference in these extraction methods can have a substantial impact on the accuracy with which the structures is classified. For this reason, we compared the classification accuracies of the two extraction methods. The cutoff method defines the “spherical” local structure of a certain bead as all beads within a cutoff radius $r_c$ centered on that bead. The count-up method defines the local structure of a certain bead by counting up to an upper limit number $N_\text{lim}$ of beads in order of proximity from that bead. The classification accuracy of the count-up method was nearly equal to that of the cutoff method. However, the count-up method lacked neighboring particles for LOP calculation when we set the $N_\text{lim}$ value above a certain level. This is because of a combination of the count-up method’s property of using $N_\text{lim}$ neighboring particles for the LOP calculation in order of shortest distance and the fact that there is a space where there were no particles under the “A or nothing” condition in which all B-blocks are removed.

Therefore, we used only the A-blocks as the input structures, and we used the cutoff method to determine the local structure. Thus the shape of the local structure was spherical. In the following sections, we reveal the results of the search for LOPs that distinguish disordered and cylindrical, cylindrical and gyroid, gyroid and lamellar, as well as cylindrical and lamellar structures.

### Disordered and cylindrical structures

Table 1 shows the classification accuracy (*c*) of LOPs that distinguish disordered and cylindrical structures on $f=0.33$. Here, we set $r_c=2.850$, 3.700, and $4.700\,\sigma _\text{u}$ based on the peak positions of the RDFs for the disordered and cylindrical structures (Fig. 1b). The first column in Table 1 is the cutoff radius $r_c$ defining the local structure size for calculating LOP values. The second and subsequent columns show LOPs with the highest classification accuracy for each definition function and their classification accuracy. The subscripts of LOPs are the internal parameters included in the definition function. For details on internal parameters, see Sect. S1 in Supplementary Materials, and Ref.^39,40^. For all LOPs, the classification accuracy tended to improve with increasing $r_c$. For the classification of disordered and cylindrical structures on $f=0.33$, the most appropriate LOP was $\overline{Q}_6^\text{S}$. The $\overline{Q}^\text{S}_6$ showed a high classification accuracy of $c=1.000$ even in the relatively small local structure size of $r_c=4.700\,\sigma _\text{u}$. The diameter of the local area ($=9.4\,\sigma _\text{u}$) was smaller than the diameter of the cylinder ($\approx 16\,\sigma _\text{u}$), the characteristic size of a cylindrical structure, but larger than the minimum thickness of the space between the two cylinders ($\approx 8.5\,\sigma _\text{u}$). The distinction between disordered and cylindrical structures using $\overline{Q}^\text{S}_6$ with $r_c=4.700\,\sigma _\text{u}$ was also performed for $f=0.32$ and $\chi N=40$. The $\overline{Q}^\text{S}_6$ with $r_c=4.700\,\sigma _\text{u}$ was able to distinguish between the two structures with the high classification accuracy of $c=1.000$ under both conditions. The diameter of the local area was larger than the minimum thickness of the space between the two cylinders for $f=0.32$, 0.33 and $\chi N=40$. Therefore, by using $\overline{Q}^\text{S}_6$ and setting $r_c=4.700\,\sigma _\text{u}$, the disordered and cylindrical structures could be classified with sufficient accuracy over a wide range on the phase diagram.

**Table 1:** Classification accuracy of LOPs for disordered and cylindrical structures on $f=0.33$.

$r_c$	LOPs													
$[\sigma _\text{u}]$							*c*$[-]$							
2.850	$\overline{A}_1^{1}$	$\overline{B}_{2,2,\pi /4}$	$\overline{C}$	$\overline{D}_{f_2,f_2,f_1}$	${F}_{f_2,f_2,1}$	$\overline{I}$	$\overline{Q}^\text{S}_{4}$	$\overline{Q}^\text{L}_{4}$	$\overline{LQ}_{4}$	$\overline{LQ}^\text{T}_{4}$	$\overline{W}^\text{S}_{4}$	$\overline{W}^\text{L}_{4}$	$\overline{LW}_{4}$	$\overline{LW}^\text{T}_{4}$
	0.975	0.964	0.814	0.915	0.728	0.967	0.974	0.972	0.699	0.684	0.573	0.560	0.580	0.554
3.700	$\overline{A}_1^{1}$	$\overline{B}_{2,2,\pi /4}$	*C*	$\overline{D}_{f_2,f_2,f_1}$	$\overline{F}_{f_1,f_1,1}$	$\overline{I}$	$\overline{Q}^\text{S}_{4}$	$\overline{Q}^\text{L}_{6}$	$\overline{LQ}_{6}$	$\overline{LQ}^\text{T}_{4}$	$\overline{W}^\text{S}_{6}$	$\overline{W}^\text{L}_{4}$	$\overline{LW}_{6}$	$\overline{LW}^\text{T}_{4}$
	0.996	0.984	0.789	0.969	0.729	0.991	0.995	0.993	0.803	0.662	0.582	0.560	0.588	0.566
4.700	$\overline{A}_2^{1}$	$\overline{B}_{2,2,\pi /4}$	*C*	$\overline{D}_{f_2,f_2,f_1}$	$\overline{F}_{f_1,f_1,1}$	$\overline{I}$	$\overline{Q}^\text{S}_{6}$	$\overline{Q}^\text{L}_{6}$	$\overline{LQ}_{6}$	$\overline{LQ}^\text{T}_{6}$	$\overline{W}^\text{S}_{8}$	$\overline{W}^\text{L}_{4}$	$\overline{LW}_{4}$	$\overline{LW}^\text{T}_{4}$
	0.999	0.991	0.797	0.976	0.813	0.999	1.000	1.000	0.913	0.702	0.592	0.610	0.594	0.658

The LOP species with the highest correct tagging rate (*c*) in each LOP series is shown for every neighboring particle selection protocol $r_\text{c}$.

### Cylindrical and gyroid structures

**Fig. 2:**
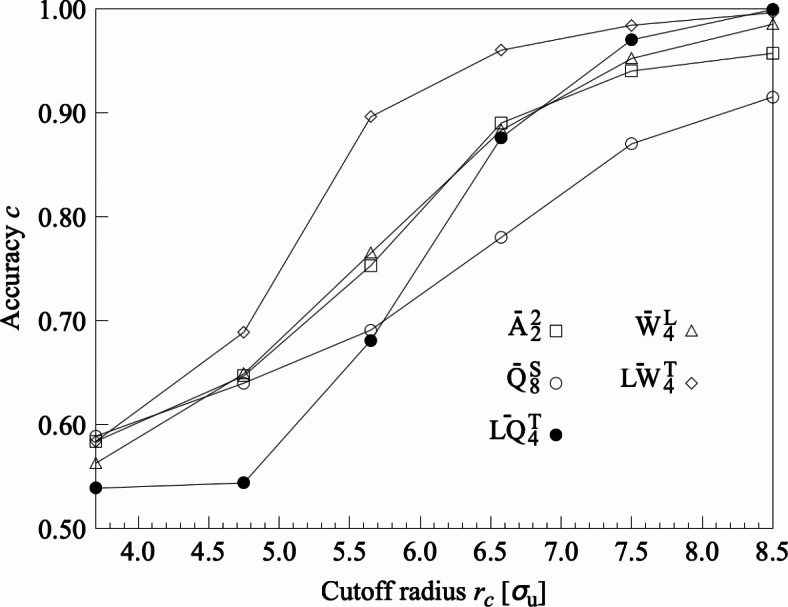
Classification accuracy (*c*) of LOPs to distinguish between cylindrical and gyroid structures on $f=0.33$. Only $\overline{A}_2^{2}$, $\overline{Q}^\text{S}_{8}$, $\overline{W}^\text{L}_{4}$, $\overline{LQ}^\text{T}_{4}$, and $\overline{LW}^\text{T}_{4}$ (validated in Table 2) are shown.

Table 2 shows the classification accuracy (*c*) of LOPs that distinguish between cylindrical and gyroid structures on $f=0.33$. Here, we set $r_c=3.700$, 4.750, 5.650, and $6.575\,\sigma _\text{u}$ based on the peak positions of the RDFs for the cylindrical and gyroid structures (Fig. 1b). Even for the $LW^\text{T}$, $LQ^\text{T}$, and *A* series, which have the highest classification accuracy among those shown in Table 2 ($c\le 0.96$), it was impossible to accurately characterize and distinguish the ordered structures with $r_c\le 6.575\,\sigma _\text{u}$. Consequently, we conducted an additional evaluation by using the $LW^\text{T}$, $LQ^\text{T}$, and *A* series with $r_c=7.500$ and $8.500\,\sigma _\text{u}$; $\overline{LQ}^\text{T}_4$ with $r_c=8.500\,\sigma _\text{u}$ can distinguish cylindrical and gyroid structures with $c=0.999$ (Fig. 2). The diameter of the local area for calculating LOP values ($=15\,\sigma _\text{u}$) was shorter than the diameter of the cylinder ($\approx 16\,\sigma _\text{u}$), while the diameter of the local area ($=17\,\sigma _\text{u}$) was longer than the diameter of the cylinder. Furthermore, using $\overline{LQ}^\text{T}_4$ with $r_c=8.500\,\sigma _\text{u}$ to distinguish cylindrical and gyroid structures on $f=0.32$ and $\chi N=40$, respectively, we distinguished the two ordered structures with high classification accuracy of $c=0.999$ and 1.000 in both conditions. The increase in classification accuracy on $\chi N=40$ corresponded to a decrease in cylinder diameter, indicating that the diameter of the local area must be set larger than the cylinder diameter for accurate classification of cylindrical and gyroid structures. Therefore, by using $\overline{LQ}^\text{T}_4$ and by defining a sufficient local structure size of $r_c=8.500\,\sigma _\text{u}$, cylindrical and gyroid structures could be classified with high accuracy over a wide range on the phase diagram.

**Table 2:** Classification accuracy of LOPs for cylindrical and gyroid structures on $f=0.33$.

$r_c$	LOPs													
$[\sigma _\text{u}]$							*c*$[-]$							
3.700	$\overline{A}_1^{1}$	$\overline{B}_{2,2,\pi /4}$	$\overline{C}$	$\overline{D}_{f_2,f_2,f_2}$	$\overline{F}_{f_1,f_2,1}$	$\overline{I}$	$\overline{Q}^\text{S}_{6}$	$\overline{Q}^\text{L}_{8}$	$\overline{LQ}_{4}$	$\overline{LQ}^\text{T}_{4}$	$\overline{W}^\text{S}_{4}$	$\overline{W}^\text{L}_{4}$	$\overline{LW}_{6}$	$\overline{LW}^\text{T}_{4}$
	0.605	0.559	0.552	0.574	0.560	0.573	0.596	0.590	0.604	0.539	0.519	0.563	0.515	0.584
4.750	$\overline{A}_2^{2}$	$\overline{B}_{2,1,\pi /4}$	$\overline{C}$	$\overline{D}_{f_2,f_2,f_2}$	$\overline{F}_{f_1,f_1,1}$	$\overline{I}$	$\overline{Q}^\text{S}_{8}$	$\overline{Q}^\text{L}_{8}$	$\overline{LQ}_{4}$	${LQ}^\text{T}_{4}$	$\overline{W}^\text{S}_{4}$	$\overline{W}^\text{L}_{4}$	$\overline{LW}_{4}$	$\overline{LW}^\text{T}_{4}$
	0.647	0.591	0.571	0.619	0.592	0.605	0.640	0.648	0.611	0.559	0.538	0.649	0.569	0.689
5.650	$\overline{A}_2^{2}$	$\overline{B}_{2,1,\pi /4}$	$\overline{C}$	$\overline{D}_{f_2,f_2,f_2}$	$\overline{F}_{f_1,f_1,1}$	$\overline{I}$	$\overline{Q}^\text{S}_{8}$	$\overline{Q}^\text{L}_{8}$	$\overline{LQ}_{4}$	$\overline{LQ}^\text{T}_{4}$	$\overline{W}^\text{S}_{4}$	$\overline{W}^\text{L}_{4}$	$\overline{LW}_{4}$	$\overline{LW}^\text{T}_{4}$
	0.753	0.631	0.590	0.677	0.631	0.643	0.691	0.635	0.589	0.681	0.565	0.765	0.615	0.896
6.575	$\overline{A}_2^{2}$	$\overline{B}_{2,1,\pi /4}$	$\overline{C}$	$\overline{D}_{f_1,f_1,f_1}$	$\overline{F}_{f_1,f_1,1}$	$\overline{I}$	$\overline{Q}^\text{S}_{8}$	$\overline{Q}^\text{L}_{6}$	$\overline{LQ}_{4}$	$\overline{LQ}^\text{T}_{4}$	$\overline{W}^\text{S}_{4}$	$\overline{W}^\text{L}_{4}$	$\overline{LW}_{6}$	$\overline{LW}^\text{T}_{4}$
	0.890	0.686	0.620	0.786	0.688	0.722	0.780	0.621	0.681	0.876	0.608	0.883	0.617	0.960

The LOP species with the highest correct tagging rate (*c*) in each LOP series is shown for every neighboring particle selection protocol $r_\text{c}$.

### Gyroid and lamellar structures

**Fig. 3:**
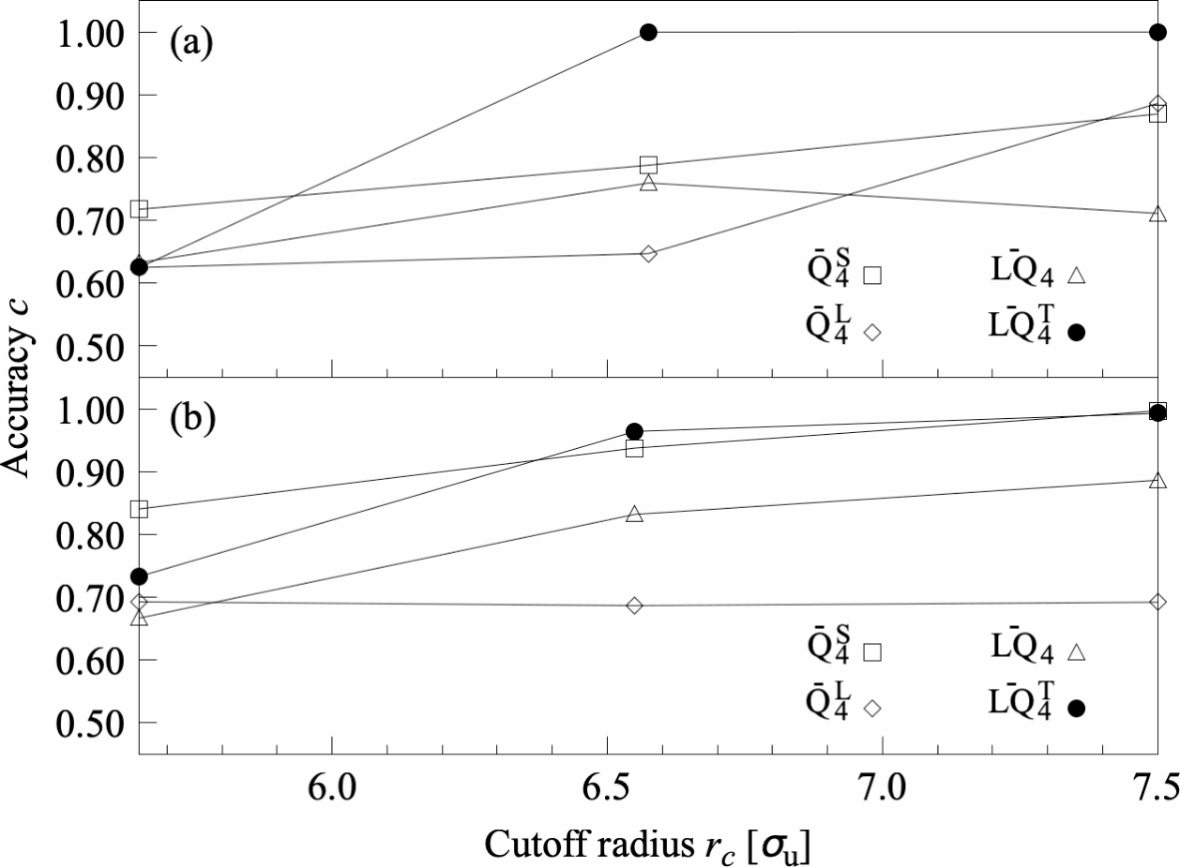
Classification accuracy (*c*) of LOPs that distinguish between the ordered structures, which include lamellar structure on $\chi N=40$. (**a**) Structural classification of gyroid and lamellar structures. (**b**) Structural classification of cylindrical and lamellar structures. Among $\overline{Q}^\text{S}_{4}$, $\overline{Q}^\text{L}_{4}$, $\overline{LQ}_{4}$, and $\overline{LQ}^\text{T}_{4}$ exhibited high robustness to $\chi N$.

Table 3 shows the classification accuracy (*c*) of LOPs distinguishing gyroid and lamellar structures on $f=0.33$. Here, we set $r_c=3.750$, 4.750, 5.650, and $6.575\,\sigma _\text{u}$ based on the peak positions of the RDFs for the gyroid and lamellar structures (Fig. 1b). When we classified gyroid and lamellar structures by setting $r_c=5.650$ and $6.575\,\sigma _\text{u}$, $\overline{LQ}^\text{T}_4$, $\overline{W}^\text{L}_4$ and $\overline{LW}^\text{T}_4$ exhibits a high classification accuracy of $c=1.000$. However, it was reported that the $\overline{W}^\text{L}$ and $\overline{LW}^\text{T}$ series could not be accurately distinguished between highly ordered molecular structures^41^. Therefore, we adopted the $\overline{LQ}^\text{T}_4$, which showed the highest classification accuracy among all LOPs. The distinction between gyroid and lamellar structures using $\overline{LQ}^\text{T}_4$ with $r_c=5.650\,\sigma _\text{u}$ was also made for $f=0.32$ and $\chi N=40$. The $\overline{LQ}^\text{T}_4$ with $r_c=5.650\,\sigma _\text{u}$ exhibited the high classification accuracy $c=1.000$ when $f=0.32$, while the accuracy was as low as $c=0.625$ when $\chi N=40$. We assumed that the decrease in classification accuracy on $\chi N=40$ was associated with an increase in the lamellar thickness. $\overline{LQ}^\text{T}_4$ accurately distinguishes between gyroid and lamella when the spherical local structure contains interfacial structures. In other words, $\overline{LQ}^\text{T}_4$ is sensitive to the difference between the interfacial structures of gyroid and lamella. When the lamellar thickness is larger than the spherical local structure, the interface structure of the lamella is less likely to be contained within the local structure. This makes it difficult to distinguish the interface structure from that of the gyroid, leading to a decrease in classification accuracy. Therefore, additional structure classification was performed by setting the local structure size for calculating LOPs (i.e., twice the cutoff radius) equal to the lamellar thickness. Figure 3a shows the classification accuracy of LOPs for the $Q^\text{S}$, $Q^\text{L}$, *LQ*, and $LQ^\text{T}$ series. The $\overline{LQ}^\text{T}_4$ with $r_c=7.500\,\sigma _\text{u}$ showed the highest classification accuracy of $c=1.000$. The fact provides strong evidence that $\overline{LQ}^\text{T}_4$ has universality to distinguish between gyroid and lamella, as long as the appropriate local structure information is provided for the LOP calculation. In other words, $\overline{LQ}^\text{T}_4$ has the ability to distinguish between gyroid and lamella over a wide area on the phase diagram.

**Table 3:** Classification accuracy of LOPs for gyroid and lamellar structures on $f=0.33$.

$r_c$	LOPs													
$[\sigma _\text{u}]$							*c*$[-]$							
3.750	$\overline{A}_4^{2}$	$\overline{B}_{2,1,0}$	$\overline{C}$	$\overline{D}_{f_2,f_2,f_2}$	$\overline{F}_{f_1,f_1,1}$	$\overline{I}$	$\overline{Q}^\text{S}_{6}$	$\overline{Q}^\text{L}_{4}$	$\overline{LQ}_{4}$	${LQ}^\text{T}_{4}$	$\overline{W}^\text{S}_{4}$	$\overline{W}^\text{L}_{4}$	$\overline{LW}_{4}$	${LW}^\text{T}_{4}$
	0.672	0.640	0.600	0.652	0.624	0.618	0.643	0.640	0.732	0.663	0.678	0.878	0.827	0.849
4.750	$\overline{A}_1^{2}$	$\overline{B}_{2,1,0}$	$\overline{C}$	$\overline{D}_{f_2,f_2,f_2}$	$\overline{F}_{f_1,f_1,1}$	$\overline{I}$	$\overline{Q}^\text{S}_{8}$	$\overline{Q}^\text{L}_{6}$	$\overline{LQ}_{4}$	$\overline{LQ}^\text{T}_{4}$	$\overline{W}^\text{S}_{4}$	$\overline{W}^\text{L}_{4}$	$\overline{LW}_{4}$	$\overline{LW}^\text{T}_{4}$
	0.773	0.704	0.640	0.714	0.665	0.673	0.715	0.760	0.862	0.957	0.779	0.993	0.982	0.992
5.650	$\overline{A}_2^{2}$	$\overline{B}_{2,2,0}$	$\overline{C}$	$\overline{D}_{f_2,f_2,f_2}$	$\overline{F}_{f_1,f_1,1}$	$\overline{I}$	$\overline{Q}^\text{S}_{8}$	$\overline{Q}^\text{L}_{6}$	$\overline{LQ}_{6}$	$\overline{LQ}^\text{T}_{4}$	$\overline{W}^\text{S}_{4}$	$\overline{W}^\text{L}_{4}$	$\overline{LW}_{6}$	$\overline{LW}^\text{T}_{4}$
	0.853	0.775	0.674	0.790	0.717	0.727	0.772	0.972	0.957	1.000	0.888	1.000	0.939	1.000
6.575	$\overline{A}_4^{2}$	$\overline{B}_{2,1,\pi /4}$	$\overline{C}$	$\overline{D}_{f_2,f_2,f_2}$	$\overline{F}_{f_1,f_1,1}$	$\overline{I}$	$\overline{Q}^\text{S}_{6}$	$\overline{Q}^\text{L}_{6}$	$\overline{LQ}_{8}$	$\overline{LQ}^\text{T}_{4}$	$\overline{W}^\text{S}_{4}$	$\overline{W}^\text{L}_{4}$	$\overline{LW}_{8}$	$\overline{LW}^\text{T}_{4}$
	0.852	0.780	0.723	0.854	0.781	0.777	0.808	1.000	0.988	1.000	0.981	1.000	0.983	1.000

The LOP species with the highest correct tagging rate (*c*) in each LOP series is shown for every neighboring particle selection protocol $r_\text{c}$.

### Cylindrical and lamellar structures

Table 4 shows the classification accuracy (*c*) of LOPs that distinguishes between cylindrical and lamellar structures on $f=0.33$. Here, we set $r_c=3.700$, 4.750, 5.650, and $6.550\,\sigma _\text{u}$ based on the peak positions of the RDFs for the cylindrical and lamellar structures (Fig. 1b). $\overline{LQ}^\text{T}_4$ with $r_c=5.650\,\sigma _\text{u}$ showed a high accuracy of $c=1.000$ in the structural classification of cylindrical and lamellar structures. The local structure size for calculating LOPs was larger than the lamellar thickness. The distinction between cylindrical and lamellar structures using $\overline{LQ}^\text{T}_4$ with $r_c=5.650\,\sigma _\text{u}$ was also made for $f=0.32$ and $\chi N=40$. $\overline{LQ}^\text{T}_4$ with $r_c=5.650\,\sigma _\text{u}$ exhibited the high classification accuracy $c=1.000$ when $f=0.32$, while the accuracy was as low as $c=0.733$ when $\chi N=40$. Therefore, additional structure classification was performed by setting the local structure size equal to the lamellar thickness. Fig. 3b shows the classification accuracy of LOPs for the $Q^\text{S}$, $Q^\text{L}$, *LQ*, and $LQ^\text{T}$ series. The $\overline{LQ}^\text{T}_4$ with $r_c=7.500\,\sigma _\text{u}$ showed the highest classification accuracy of $c=0.993$. The fact implies that $\overline{LQ}^\text{T}_4$ has the universality to distinguish between cylindrical and lamellar structures, i.e., the ability to distinguish between the two structures over the wide area on the phase diagram.

**Table 4:** Classification accuracy of LOPs for cylindrical and lamellar structures on $f=0.33$.

$r_c$	LOPs													
$[\sigma _\text{u}]$							*c*$[-]$							
3.700	$\overline{A}_1^{1}$	$\overline{B}_{2,1,0}$	$\overline{C}$	$\overline{D}_{f_2,f_2,f_2}$	$\overline{F}_{f_1,f_1,1}$	$\overline{I}$	$\overline{Q}^\text{S}_{6}$	$\overline{Q}^\text{L}_{8}$	$\overline{LQ}_{4}$	${LQ}^\text{T}_{4}$	$\overline{W}^\text{S}_{4}$	$\overline{W}^\text{L}_{4}$	$\overline{LW}_{4}$	${LW}^\text{T}_{4}$
	0.734	0.660	0.618	0.686	0.648	0.664	0.718	0.678	0.784	0.685	0.648	0.823	0.813	0.831
4.750	$\overline{A}_2^{2}$	$\overline{B}_{2,1,0}$	$\overline{C}$	$\overline{D}_{f_2,f_2,f_2}$	$\overline{F}_{f_1,f_1,1}$	$\overline{I}$	$\overline{Q}^\text{S}_{8}$	$\overline{Q}^\text{L}_{4}$	$\overline{LQ}_{4}$	$\overline{LQ}^\text{T}_{4}$	$\overline{W}^\text{S}_{4}$	$\overline{W}^\text{L}_{4}$	$\overline{LW}_{4}$	$\overline{LW}^\text{T}_{6}$
	0.847	0.733	0.677	0.783	0.719	0.741	0.810	0.735	0.919	0.963	0.741	0.976	0.977	0.971
5.650	$\overline{A}_4^{2}$	$\overline{B}_{2,1,0}$	$\overline{C}$	$\overline{D}_{f_2,f_2,f_2}$	$\overline{F}_{f_1,f_1,1}$	$\overline{I}$	$\overline{Q}^\text{S}_{8}$	$\overline{Q}^\text{L}_{4}$	$\overline{LQ}_{6}$	$\overline{LQ}^\text{T}_{4}$	$\overline{W}^\text{S}_{4}$	$\overline{W}^\text{L}_{4}$	$\overline{LW}_{6}$	$\overline{LW}^\text{T}_{6}$
	0.978	0.802	0.729	0.886	0.797	0.822	0.902	0.962	0.951	1.000	0.831	0.995	0.919	0.998
6.550	$\overline{A}_2^{2}$	$\overline{B}_{2,1,\pi /2}$	$\overline{C}$	$\overline{D}_{f_2,f_2,f_2}$	$\overline{F}_{f_1,f_1,1}$	$\overline{I}$	$\overline{Q}^\text{S}_{8}$	$\overline{Q}^\text{L}_{6}$	$\overline{LQ}_{8}$	$\overline{LQ}^\text{T}_{4}$	$\overline{W}^\text{S}_{4}$	$\overline{W}^\text{L}_{6}$	$\overline{LW}_{8}$	$\overline{LW}^\text{T}_{6}$
	1.000	0.888	0.790	0.987	0.893	0.934	0.975	1.000	0.983	1.000	0.941	1.000	0.977	1.000

The LOP species with the highest correct tagging rate (*c*) in each LOP series is shown for every neighboring particle selection protocol $r_\text{c}$.

## Conclusion

In this study, to accurately characterize microphase-separated structures of diblock copolymers, we searched for LOPs that accurately distinguish adjacent structures on the phase diagram. We used $f=0.32$, 0.33, and $\chi N=40$ as the straight lines on the phase diagram where four representative microphase-separated structures (i.e., disorder, cylinder, gyroid, and lamella) occur. $f=0.33$ was especially important because of the large area on the phase diagram for each structure and its correspondence with the change in structural order with temperature. We created four microphase-separated structures by careful backmapping from the continuum model to the KGBS model, and we used the machine learning software MALIO to evaluate the structural classification performance of the 186 LOP candidates. We used the structure of the A-blocks as the minimum structural information to be input into MALIO. MALIO calculated the numerical values of all LOP candidates for the input structures to create a dataset, and then performed supervised machine learning to select the best LOPs in a fast and systematic manner.

In general, $\overline{LQ}^\text{T}_4$ exhibited superior performance in distinguishing between ordered structures. In other words, it gave the smallest local structure area for LOP calculation that achieved high classification accuracy. On $f=0.33$, lamellar and gyroid, cylindrical and gyroid, and cylindrical and lamellar structures were classified with high accuracy using $\overline{LQ}^\text{T}_4$ of $r_c=5.650$, 8.500, and $5.650\,\sigma _\text{u}$, respectively. The cylindrical and lamellar structures are not adjacent on the phase diagram on $f=0.33$, but they can be adjacent under other conditions, hence the classification was performed. $\overline{Q}^\text{S}_6$ of $r_c=4.700\,\sigma _\text{u}$ was superior in distinguishing cylindrical structure from adjacent disordered structure. These results were similar on $f=0.32$, suggesting the high robustness of the LOPs selected by MALIO to *f*. Furthermore, on $\chi N=40$, the LOPs selected were exactly the same as in the constant condition of *f*. The values of $r_c$ were also the same, except for the classifications containing lamellar structure. For the classification including lamellar structure, increasing $r_c$ to approximately half of the lamellar thickness ($7.5\,\sigma _\text{u}$) resulted in a classification accuracy of $c=0.993$ or better for $\overline{LQ}^\text{T}_4$. Therefore, the LOPs selected by MALIO are highly robust to $\chi N$. The LOPs proposed in this study might be applicable to a wide area on the phase diagram.

The minimum local area size required for the LOPs selected by MALIO to achieve their classification performance seems to be closely related to the characteristic size of microphase-separated structures. For structure classification including disordered structure, high classification accuracy was achieved by defining a relatively small local structure area. This means that whether the blocks form domains or are uniformly distributed can be determined with less information contained in the local structure area. The accurate classification of cylindrical and gyroid structures required the definition of a local structure area of $r_c=8.500\,\sigma _\text{u}$, which exceeds a maximum diameter of the cylindrical structure of approximately $16\,\sigma _\text{u}$. The accurate classification of cylindrical and lamellar, or gyroid and lamellar structures required the definition of a local structure area of $r_c=7.500\,\sigma _\text{u}$, which corresponds to a maximum lamellar thickness of about $15\,\sigma _\text{u}$. This means that the accurate distinction between highly ordered domains requires information contained in the local structure area equivalent to a size representative of the structure of domain. Although it is difficult to easily measure the domain size of gyroid structure, we obtained high classification accuracy by defining the local structure area based on the characteristic domain size of lamellar or cylindrical structures.

Overall, the strategy of systematically screening a large number of LOP candidates was effective in finding LOPs that distinguish microphase-separated structures. Whether the proposed LOPs can describe changes in local order structure during phase transitions will need to be demonstrated in future studies, such as by conducting careful structural transition simulations. However, the LOPs proposed in this study can at least reliably distinguish the ordered structure before and after the phase transition, and is therefore a promising first candidate for parameters that describe the details of the phase transition. The introduction of a good quality LOP into the Ginzburg-Landau equation may lead to a detailed description of the mesoscale ordered structure transition associated with phase transitions. Furthermore, since the LOP is determined from the molecular-scale ordered structures, it may lead to an understanding of the formation process of the multiscale ordered structure. Changes in ordered structure and formation processes of block copolymers are typical problems that are not easily accessible experimentally. It is expected that LOPs discovered in a scheme similar to this study will help to solve these problems, leading to more precise control of pattern formation, etc. at the experimental level.

## Methods

**Fig. 4:**
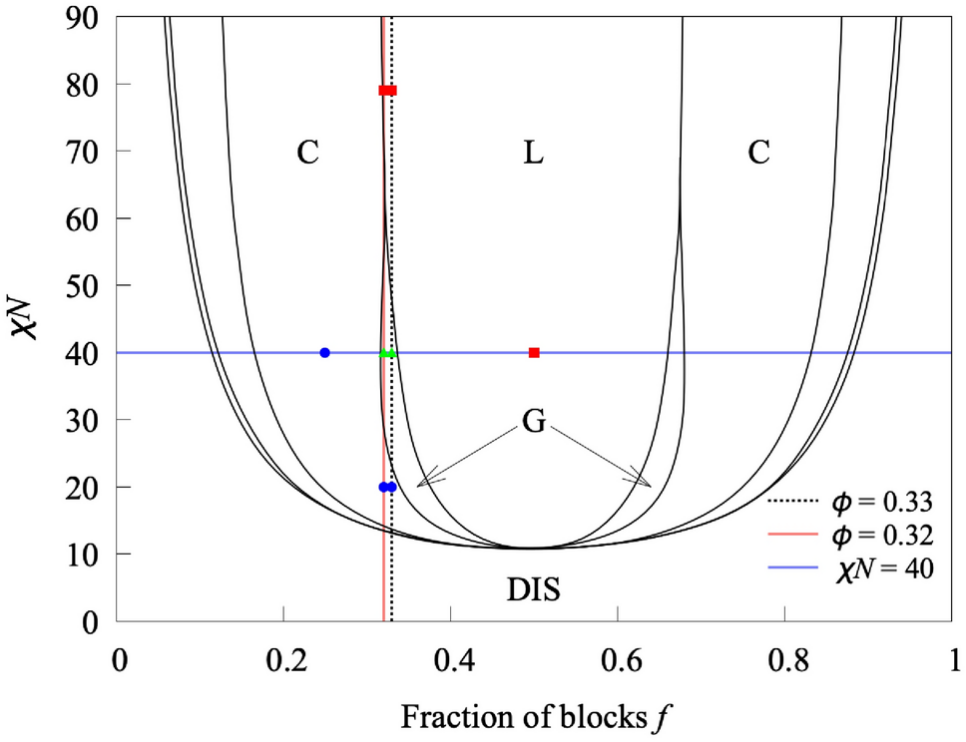
Phase diagram of diblock copolymers depicted with data from Ref.^18^. (DIS) Disordered, (C) cylindrical, (G) gyroid, (L) lamellar phases. The black dotted, red solid, and blue solid lines are constant conditions with $f=0.33$, $f=0.32$, and $\chi N=40$, respectively. The blue, green, and red points represent cylindrical, gyroid, and lamellar structures, respectively, sampled in this work. We created all the microphase-separated structures corresponding to the points by backmapping from the continuum model to the KGBS model.

A*n*B*m* diblock copolymers consisting of two types of polymer blocks (A and B) were prepared by backmapping from the continuum model to the KGBS model. The number of polymer segments in the A- and B-blocks of one polymer chain is indicated by *n* and *m*, respectively, and the fraction of block $f = n/(n+m) = n/N$. It is clear that the four microphase-separated structures (disorder, cylinder, gyroid, and lamella) exist on the lines $f=0.32$, 0.33, and $\chi N=40$ shown in Fig. 4. For $f=0.32$ and 0.33, disordered, cylindrical, gyroid, and lamellar structures were created by setting $\chi N$ to 0, 20, 40, and 79, respectively. Note that *N* was set to 125 and 120 for $f=0.32$ and 0.33, respectively. Recall that when *f* is fixed, the variation of $\chi N$ corresponds to a temperature increase or decrease. For $\chi N=40$, disorder, cylindrical and lamellar structures were created by setting *f* to 0.05, 0.25 and 0.50, respectively, while setting $N=120$. Note that the gyroid structure is at the intersection of the lines $f=0.33$ and $\chi N=40$, so the gyroid structure created for the line $f=0.33$ was used. In the next subsection, the procedure for creating the structures with the continuum model will be explained, using the condition $f=0.33$ as an example.

### Microphase-separated structures calculated from continuum models

**Fig. 5:**
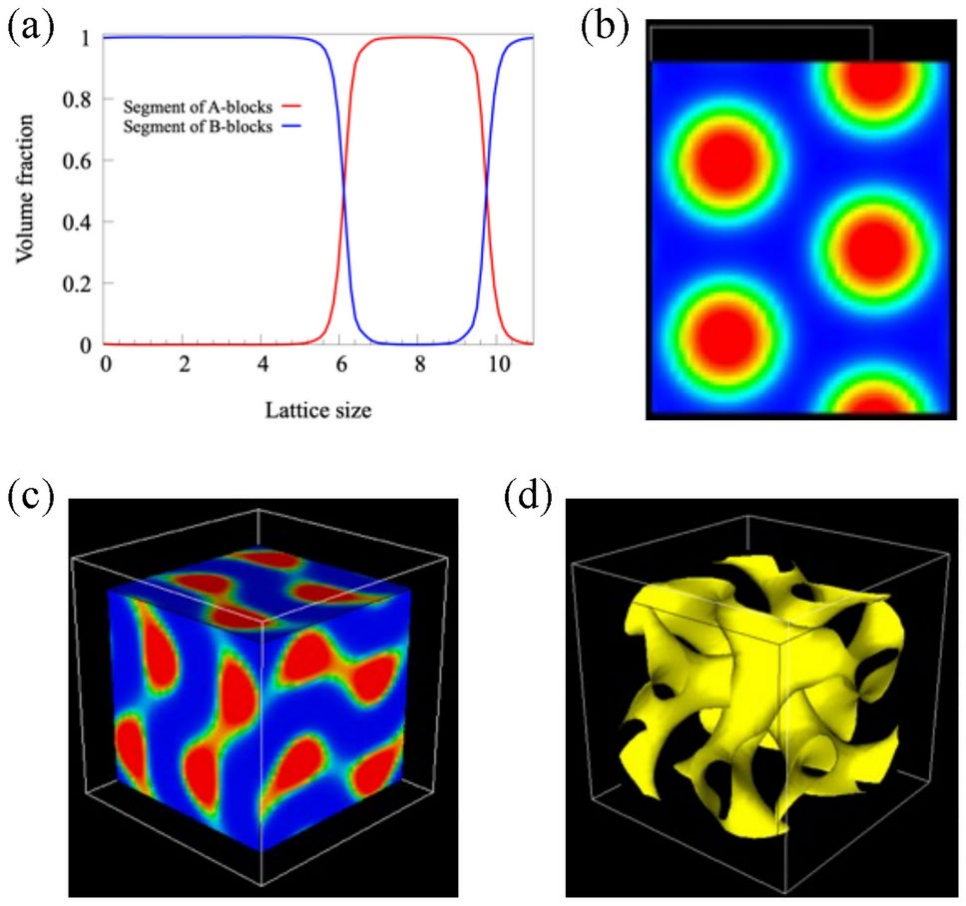
Segment profiles of microphase-separated structures calculated with SUSHI. (**a**) 1D profile of lamella, (**b**) 2D profile of cylinder, (**c,d**) 3D profile of gyroid. Red and blue are the segment profiles of the A- and B-blocks, respectively. (**d**) Surface view of the interface between the A- and B-blocks. Figure (**b–d**) were generated with SUSHI [Version: 11.0 Revision 200530, URL:https://octa.jp/].

First, to obtain the equilibrium structure of the AB block copolymer corresponding to each point shown in Fig. 4, the segment profile of each structure was calculated with Simulation Utilities for Soft and Hard Interfaces (SUSHI)^43^ developed by Honda et al. SUSHI places polymer chains in a lattice space and calculates segment profiles that minimize the free energy of polymers. The segment profile of lamellar structure was calculated by defining a one-dimensional (1D) lattice (Fig. 5a). Here, red and blue represent the profiles of the polymer segments of the A- and B-blocks, respectively. By defining two-dimensional (2D) and three-dimensional (3D) lattices, segment profiles of cylindrical and gyroid structures were calculated as well (Fig. 5b–d). These segment profiles were then backmapped to the KGBS model with coarse-grained molecular dynamics program by Nagoya Cooperation (COGNAC)^28^ developed by Aoyagi et al.

### Backmapping from continuum model to Kremer–Grest bead spring model

Using the DBMC method implemented in COGNAC, the segment profiles of cylindrical, gyroid, and lamellar equilibrium structures were efficiently backmapped to the KGBS model. The DBMC method sequentially places KG beads of polymer chains based on the Monte Carlo method weighted by the segment profiles. The number of KG beads, *N*, was set to 120 to clearly represent the polymer chains of the A40B80 diblock copolymer. The number of molecules $M = 1940$, mass $m=1.0\,m_\text{u}$, and density $\rho =0.90\,m_\text{u}/\sigma _\text{u}^3$ were set. The units of energy, length, mass, and time are represented by $\epsilon _\text{u}$, $\sigma _\text{u}$, $m_\text{u}$, and $\tau _\text{u}$ ($\tau _\text{u}=\sqrt{m_\text{u}\sigma ^2/\epsilon _\text{u}}$), respectively. The unit of temperature *T* is $\epsilon _\text{u}/k_\text{B}$, where $k_\text{B}$ is the Boltzmann constant. The initial placement of KG beads that comprise the lamellar structure was achieved by scaling the 1D lattice size of the continuum model to the unit cell size of the KGBS model in the z-direction by the DBMC method. The unit cell sizes in the x- and y-directions were calculated from *M* and $\rho$. The initial placement of KG beads comprising the gyroid and cylindrical structures was achieved similarly. For disordered structures, KG beads were placed randomly. The intermolecular interactions are represented by Lennard–Jones (LJ) potential $U^{\text{LJ}}$, and the intramolecular interactions are represented by a combination of the finite extensible nonlinear elastic (FENE) potential $U^{\text{FENE}}$ and $U^{\text{LJ}}$.

1 $$\begin{aligned} U^{\text{LJ}}_{ij}=4\epsilon _{ij}\left[ \left( \frac{\sigma _{ij}}{r_{ij}}\right) ^{12}-\left( \frac{\sigma _{ij}}{r_{ij}}\right) ^{6}\right] , \end{aligned}$$

2 $$\begin{aligned} U^{\text{FENE}}_{ij}=-\frac{1}{2}KR_0^2\ln \left[ 1-\left( \frac{r_{ij}}{R_0}\right) ^2\right] , \end{aligned}$$

where the subscripts *i* and *j* denote the index of KG beads. The parameter $\sigma _{ij}$ was set to $1.0\,\sigma _\text{u}$ regardless of the combination of block types determined by the *i*,*j* pairs. On the other hand, the parameter $\epsilon _{ij}$ was set to $1.0\,\epsilon _\text{u}$ when *i* and *j* were the same block type, and $1.0\,\epsilon _\text{u} - \delta \epsilon$ when they were different block types. $\delta \epsilon$ is a parameter for adjusting the miscibility between the A- and B-blocks; and was set to 0.00, 0.10, 0.15 and $0.50\,\epsilon _\text{u}$ for the disordered, cylindrical, gyroid, and lamellar structures, respectively. The $\delta \epsilon$ values were determined by creating lamellar structures of A50B50 diblock copolymer on $\chi N=20$, 40, 79 and comparing the interface profiles in the continuum and KGBS models. The LJ interactions were truncated at $2.5\,\sigma _\text{u}$. The maximum extent of the bond $R_0=1.5\,\sigma _\text{u}$, and the spring constant $K=30\,\epsilon _\text{u}/\sigma _\text{u}^2$ were set. The velocity verlet method was used for numerical integration, and the time increment in the time evolution was set to $0.005\,\tau _\text{u}$. Coarse-grained molecular simulations were performed with the Large-scale Atomic/Molecular Massively Parallel Simulator (LAMMPS)^44^.

**Fig. 6:**
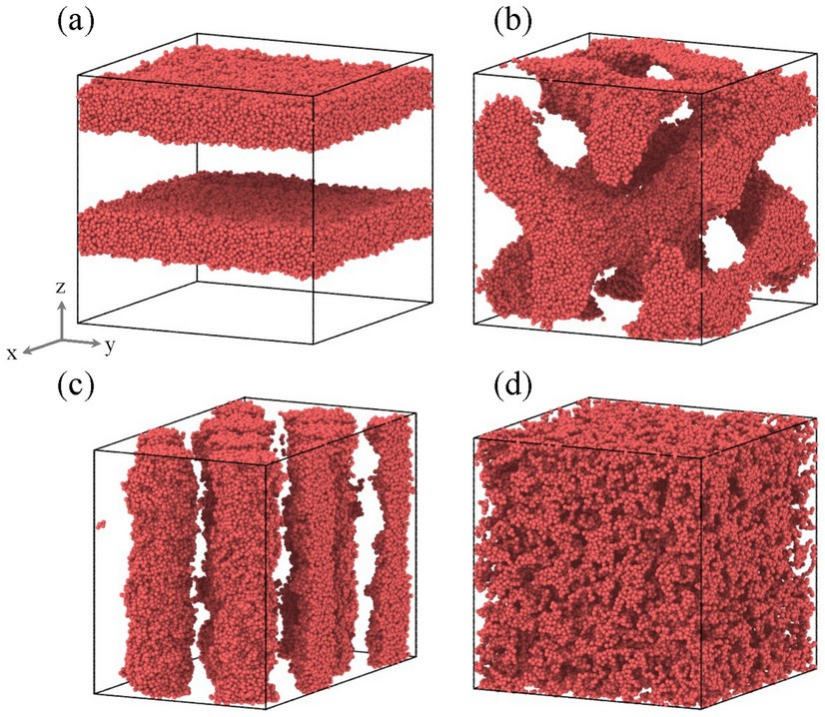
Microphase-separated structures of KGBS model on $f=0.33$. (**a**) Lamellar structure, $65\,\sigma _\text{u}\times 65\,\sigma _\text{u}\times 62\,\sigma _\text{u}$, (**b**) gyroid structure, $64\,\sigma _\text{u}\times 64\,\sigma _\text{u}\times 64\,\sigma _\text{u}$, (**c**) cylindrical structure, $84\,\sigma _\text{u}\times 49\,\sigma _\text{u}\times 63\,\sigma _\text{u}$, (**d**) disordered structure, $64\,\sigma _\text{u}\times 64\,\sigma _\text{u}\times 64\,\sigma _\text{u}$. The molecular structures consist of beads in the A- and B-blocks, but the beads in the B-blocks are not shown. The figures were generated with Open Visualization Tool (OVITO)^45^ [Version: 2.9.0, URL: https://www.ovito.org/]..

The DBMC method places KG beads with fixed bond length between beads, and therefore whether all polymer chains form a natural chain conformation is unclear. Therefore, to stabilize the conformation, relaxation calculations were performed under *NVT* ($T=1.0\epsilon _\text{u}/k_\text{B}$) at $6.0\times 10^4\,\tau _\text{u}$. The constant bead number, volume, and temperature (*NVT*) was performed by the Nose–Hoover method. Next, relaxation calculations were performed under *NPT* ($T=1.0\epsilon _\text{u}/k_\text{B}$, *P*) at $1.5\times 10^6\,\tau _\text{u}$. The constant bead number, pressure, and temperature (*NPT*) were performed by the Parrinello–Rahman method. The structures thoroughly relaxed by the *NPT* simulations (Fig. 6) were selected as the structures that should be input into MALIO.

### MALIO

MALIO is a machine learning package that identifies local structures hidden in complex structures based on LOPs^39^. MALIO implements and templates promising concepts of LOPs including algorithms, and takes proper account of the homogeneity of the dataset. When the structures to be distinguished are input into MALIO, a data set of values of a large number of candidate parameters are generated based on the templates (i.e., LOP definition functions). The data set is processed by a strategic scheme implemented in MALIO to select the LOP that best distinguishes the structures. The original versions of the 14 LOP definition functions considered in this study are outlined below, but note that the LOPs implemented in MALIO have been modified to exploit more of the potential of the original definition functions (see Section S1 of Supplementary Materials for details on the definition functions actually implemented). The neighborhood parameter *A* was developed by Honeycutt and Andersen^46,47^, and by Radhi and Behdinan^48^, to characterize the crystal structure of the LJ fluid based on the distance between the pair particles and neighbor particles. The bond-angle order parameter *B* was used by Ackland and Jones^49^ to identify dislocation defects in colloidal suspensions, based on the bond angle between the central particle and its neighbors. The centrosymmetry parameter *C* was used by Kelchner et al.^50^ to analyze dislocations and defects on metal surfaces, based on the distance between the central particle and its neighbors. The neighbor distance parameter *D* was used by Stukowski^51^ to identify the crystal structure at grain boundaries by introducing a scale factor related to the neighbor distances. The angular Fourier series parameter *F* was developed by Bartok and coworkers^52,53^ to analyze potential energy surfaces in bulk crystals and in silicon, based on periodic properties of the structure. The tetrahedral order parameter *I* was developed by Chau and Hardwick^54,55^ to evaluate tetrahedral configurations of molecules; and was applied to water, methane, as well as LJ fluids. The bond-orientational order parameters $Q^\text{S}$ and $W^\text{S}$, based on spherical harmonic functions, were originally developed by Steinhardt and coworkers^56^ to quantitatively evaluate orientational order in supercooled liquids as well as metallic glasses. The modified bond-orientational order parameters $Q^\text{L}$ and $W^\text{L}$, developed by Lechner and Dellago^57^, were used to distinguish between crystals and supercooled liquids in LJ fluids by locally averaging the spherical harmonic function term. The alternative bond-orientational order parameters *LQ* and *LW*, for which the spherical harmonic function term was normalized, were used for analyzing local molecular structures in ice nucleation, growth, or melting^58,59^. The modified alternative bond-orientational order parameters $LQ^\text{T}$ and $LW^\text{T}$, locally averaged over the normalized spherical harmonic function of *LQ* and *LW*, were first devised in Ref.^39^ and implemented in MALIO. Considering the internal parameters included in each definition function, the total number of candidate LOPs in this work was 186 (see Sect. S1 for details on internal parameters in Supplementary Materials).

**Fig. 7:**
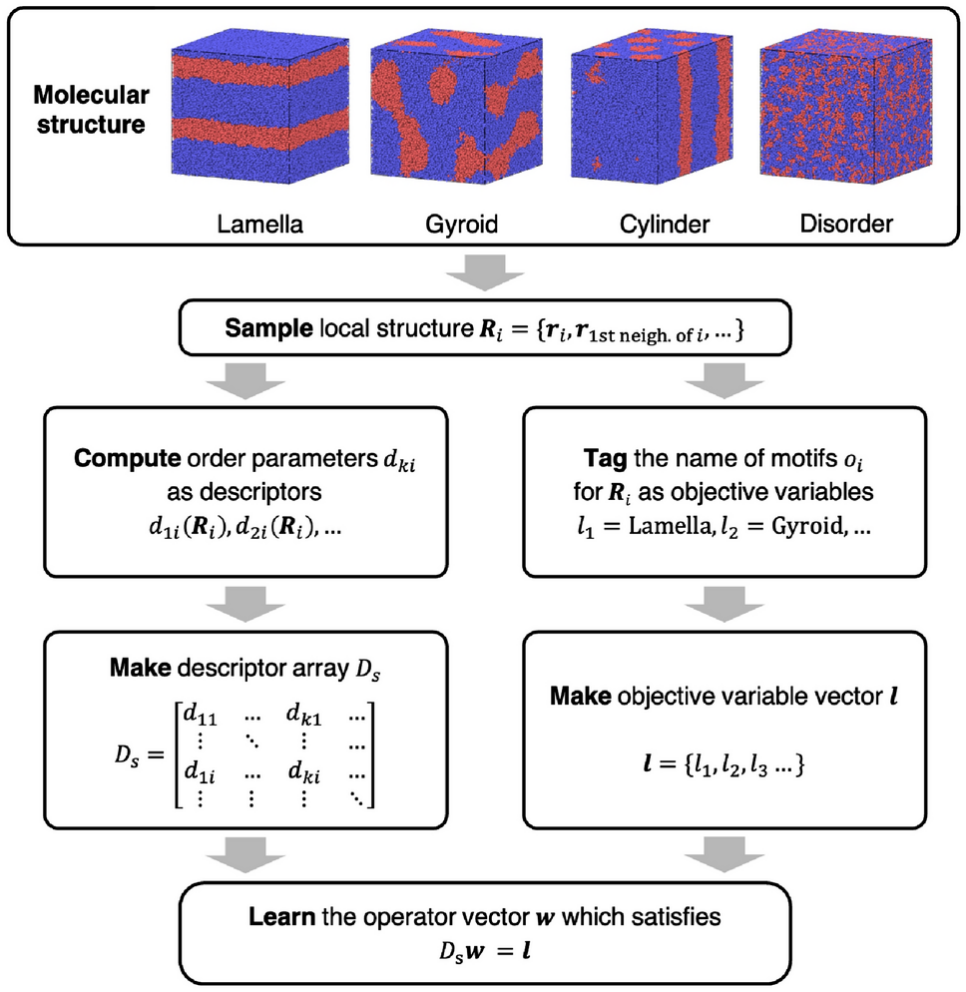
Procedure for searching LOPs by MALIO.

The procedure for searching for the LOPs using MALIO is shown in Fig. 7. The 3D particle coordinates of two adjacent structures (disorder and cylinder, cylinder and gyroid, gyroid and lamella, as well as cylinder and lamella) on the phase diagram shown in Fig. 4 were input into MALIO. From the inputs, the local structures needed to calculate the LOP values were extracted in accordance with the cutoff or count-up method. The cutoff method defines the local structure of bead *i* as all beads within a cutoff radius $r_c$ centered at bead *i*. The count-up method defines the local structure of bead *i* by counting up to an upper limit number $N_\text{lim}$ of beads in order of proximity from bead *i*. For all local structures, 186 LOP values were calculated and stored as a data set along with the corresponding input structure names. Supervised machine learning was performed to learn the correspondence between LOP values and structure names. The random forest method^60^ was used as the classifier, the number of decision trees was set to 100, and the maximum depth was set to 10. All other parameter settings were set to the Scikit-learn defaults. Furthermore, k-fold cross validation, implemented in Scikit-learn^61^, was performed five times to confirm that there was no overlearning. MALIO estimates the classification accuracy of LOPs $c=Z_{\text{correct}}/Z_{\text{total}}$; where $Z_{\text{correct}}$ is the number of correct structure names predicted by LOPs, and $Z_{\text{total}}$ is the number of all structure names input to MALIO.

## Supplementary Information

Supplementary Information.

## Data Availability

The data that support the findings of this study are available from the corresponding author upon reasonable request.
