# Supplementary material for: Distinguish microphase-separated structures of diblock copolymers using local order parameters
Source: Sci Rep. 2024 Oct 13;14:23908. doi: 10.1038/s41598-024-74525-y (PMC11471776; doi:10.1038/s41598-024-74525-y)
Supplement: Supplementary file 1 — Supplementary Information. [file 41598_2024_74525_MOESM1_ESM.docx]

Supplementary Materials for

**Distinguish microphase-separated structures of diblock copolymers using local order parameters**

Fumiki Takano *^a, b^*, Masaki Hiratsuka *^a^*, Kazuaki Z. Takahashi *^b, *^*

*^a^ Kogakuin University,1-24-2 Nishi-Shinjuku, Tokyo, 163-8677, Japan*

*^b^ National Institute of Advanced Industrial Science and Technology (AIST),*

*Research Center for Computational Design of Advanced Functional Materials,*

*Central 2, 1-1-1 Umezono, Tsukuba, Ibaraki, 305-8568, Japan*

^*^ To whom correspondence should be addressed; E-mail: kazu.takahashi@aist.go.jp

**S1. Definition functions of local order parameters (LOPs)**

Fourteen definition functions ($A, B, C, D, F, I, Q^{S}, W^{S}, Q^{L}, W^{L}, LQ, LW, LQ^{T}, LW^{T}$) were adopted from among those implemented in MALIO[1], and a total of 186 LOPs were investigated, considering the internal parameters included in each definition function. The neighborhood parameter $A$ is expressed as

$$\begin{aligned} A_{M}^{1}\left( i \right)=\frac{1}{N}\sum_{j\in\boldsymbol{N}_{b}\left( i \right)} \left| \sum_{k\in\boldsymbol{N}_{b}\left( i,j \right)} \left( \boldsymbol{r}_{ik}+\boldsymbol{r}_{jk} \right) \right|^{2}\#\left( 1 \right) \end{aligned}$$

$$\begin{aligned} A_{M}^{2}\left( i \right)=\frac{1}{N}\sum_{j\in\boldsymbol{N}_{b}\left( i \right)} \left| \sum_{k\in\boldsymbol{N}_{b}\left( i,j \right)} \left( \boldsymbol{r}_{ij}+\boldsymbol{r}_{kj} \right) \right|^{2}\#\left( 2 \right) \end{aligned}$$

$$\begin{aligned} A_{M}^{3}\left( i \right)=\frac{1}{N}\left| \sum_{j\in\boldsymbol{N}_{b}\left( i \right)} \sum_{k\in\boldsymbol{N}_{b}\left( i,j \right)} \left( \boldsymbol{r}_{ij}+\boldsymbol{r}_{kj} \right) \right|^{2}\#\left( 3 \right) \end{aligned}$$

where $\boldsymbol{N}_{b}\left( i \right)$ is an array that stores the identifiers of neighbor particles of particle $i$ in order of decreasing distance from particle $i$, $\boldsymbol{N}_{b}\left( i, j \right)$ is an array of identifiers of mutually neighboring particles of particles $i$ and $j$, $N$ and $M$ denote the number of elements in arrays $\boldsymbol{N}_{b}\left( i \right)$ and $\boldsymbol{N}_{b}\left( i, j \right)$, and $\boldsymbol{r}_{ij}$ is a vector from particle $j$ to particle $i$. We consider $M=1$, $2$, and $4$. Local averaging of LOP value implemented in MALIO for all 14 definition functions was also investigated. The local averaging process is an operation in which the LOP value of one particle is averaged with the LOP values of its neighboring particles. Locally averaged LOPs are denoted by superscript bars. The locally averaged neighborhood parameter $\bar{A}$ is defined as

$$\begin{aligned} \bar{A}_{M}^{\mathrm{var}}\left( i \right)=\frac{1}{N+1}\sum_{j\in{\tilde{\boldsymbol{N}}}_{b}\left( i \right)} A_{M}^{\mathrm{var}}\left( j \right)\#\left( 4 \right) \end{aligned}$$

where the superscript “var” indicates the variation from 1 to 3, and ${\tilde{\boldsymbol{N}}}_{b}\left( i \right)$ is an array of identifiers of neighboring particles and particle $i$. The local averaging was based on ${\tilde{\boldsymbol{N}}}_{b}$ and did not lead to an increase in the effective number of neighboring particles.

The bond-angle order parameter $B$ is expressed as

$$\begin{aligned} B_{n_{1}, n_{2},\phi}\left( i \right)=\frac{1}{N\left( N-1 \right)/2}\sum_{j>k\in\boldsymbol{N}_{b}\left( i \right)} f\left( \theta_{jik} \right)\#\left( 5 \right) \end{aligned}$$

$$\begin{aligned} f\left( \theta_{jik} \right)=\cos^{n_{1}} \left( n_{2}\theta_{jik}+\phi\right)\#\left( 6 \right) \end{aligned}$$

where $\theta_{jik}$ is the angle between $\boldsymbol{r}_{ij}$ and $\boldsymbol{r}_{ik}$, $n_{1}$ and $n_{2}$ are positive integers, and $\phi$ is an offset angle. We consider $n_{1} = 2$; $n_{2} = 1$, and $2$; and $\phi=0, \pi/4$, and $\pi/2$. The locally averaged bond-angle order parameter $\bar{B}$ is defined as

$$\begin{aligned} \bar{B}_{n_{1}, n_{2},\phi}\left( i \right)=\frac{1}{N+1}\sum_{j\in{\tilde{\boldsymbol{N}}}_{b}\left( i \right)} B_{n_{1}, n_{2},\phi}\left( j \right)\#\left( 7 \right) \end{aligned}$$

The centrosymmetry parameter C is expressed as

$$\begin{aligned} C\left( i \right)=\sum_{j\in\boldsymbol{N}_{b}^{'}\left( i \right)} \left| \boldsymbol{r}_{ij}+\boldsymbol{r}_{ik} \right|^{2}\#\left( 8 \right) \end{aligned}$$

where $\boldsymbol{N}_{b}^{'}\left( i \right)$ is an array of identifiers of neighbor particles of half of $\boldsymbol{N}_{b}\left( i \right)$ in order of nearest-neighbor from particle $i$, $N'$ denotes the number of elements in array $\boldsymbol{N}_{b}^{'}\left( i \right)$, and $k$ satisfies the relation$k=j+N-N'$. The locally averaged centrosymmetry parameter $\bar{C}$ is defined as

$$\begin{aligned} \bar{C}\left( i \right)=\frac{1}{N+1}\sum_{j\in{\tilde{\boldsymbol{N}}}_{b}\left( i \right)} C\left( j \right)\#\left( 9 \right) \end{aligned}$$

Table S1 Functions represented by $f_{\alpha}$, $f_{\beta}$, and $f_{\gamma}$

| Function | Formula |
| --- | --- |
| $f_{1}$ | $r$ |
| $f_{2}$ | $1/r$ |

The neighbor distance parameter $D$ is expressed as

$$\begin{aligned} D_{f_{\alpha},f_{\beta},f_{\gamma}}\left( i \right)=\frac{1}{N\left( N-1 \right)/2}\sum_{j>k\in\boldsymbol{N}_{b}\left( i \right)} f_{\alpha}\left( \boldsymbol{r}_{ij} \right)f_{\beta}\left( \boldsymbol{r}_{ik} \right)f_{\gamma}\left( \boldsymbol{r}_{jk} \right)\#(10) \end{aligned}$$

where $f_{\alpha}, f_{\beta}$, and $f_{\gamma}$ denote the scale factor functions of the distance $r$. The specific forms of $f_{\alpha}, f_{\beta}$, and $f_{\gamma}$ correspond to the functions listed for each subscript of the parameters in Table S1. The locally averaged neighbor distance parameter $\bar{D}$ is defined as

$$\begin{aligned} \bar{D}_{f_{\alpha},f_{\beta},f_{\gamma}}\left( i \right)=\frac{1}{N+1}\sum_{j\in{\tilde{\boldsymbol{N}}}_{b}\left( i \right)} D_{f_{\alpha},f_{\beta},f_{\gamma}}\left( j \right)\#\left( 11 \right) \end{aligned}$$

The angular Fourier series parameter $F$ is expressed as

$$\begin{aligned} F_{f_{\alpha},f_{\beta},a}\left( i \right)=\frac{1}{N\left( N-1 \right)/2}\sum_{j>k\in\boldsymbol{N}_{b}\left( i \right)} f_{\alpha}\left( \min\left( \boldsymbol{r}_{ij},\boldsymbol{r}_{ik} \right) \right)f_{\beta}\left( \max\left( \boldsymbol{r}_{ij},\boldsymbol{r}_{ik} \right) \right)\cos\left( a\theta_{jik} \right)\#\left( 12 \right) \end{aligned}$$

where $a$ is an angular factor. We consider $a=1$. The locally averaged angular Fourier series parameter $\bar{F}$ is defined as

$$\begin{aligned} \bar{F}_{f_{\alpha},f_{\beta},a}\left( i \right)=\frac{1}{N+1}\sum_{j\in{\tilde{\boldsymbol{N}}}_{b}\left( i \right)} F_{f_{\alpha},f_{\beta},a}\left( j \right)\#\left( 13 \right) \end{aligned}$$

The tetrahedral order parameter $I$ is expressed as

$$\begin{aligned} I\left( i \right)=1-\frac{3}{8}\sum_{j>k\in\boldsymbol{N}_{b}\left( i \right)} \left[ \cos\left( \theta_{jik} \right)+1/3 \right]^{2}\#\left( 14 \right) \end{aligned}$$

The locally averaged tetrahedral order parameter $\bar{I}$ is defined as

$$\begin{aligned} \bar{I}\left( i \right)=\frac{1}{N+1}\sum_{j\in{\tilde{\boldsymbol{N}}}_{b}\left( i \right)} I\left( j \right)\#\left( 15 \right) \end{aligned}$$

The bond-orientational order parameters $Q^{S}$ and $W^{S}$ are expressed as

$$\begin{aligned} Q_{l}^{S}\left( i \right)=\sqrt{\frac{4\pi}{2l+1}\sum_{m=-l}^{l} \left| q_{lm}\left( i \right) \right|^{2}}\#\left( 16 \right) \end{aligned}$$

$$\begin{aligned} W_{l}^{S}\left( i \right)=\sum_{m_{1}+m_{2}+m_{3}=0} \left( \begin{matrix} l & l & l \\ m_{1} & m_{2} & m_{3} \end{matrix} \right)q_{lm_{1}}\left( i \right)q_{lm_{2}}\left( i \right)q_{lm_{3}}\left( i \right)/\left( \sum_{m=-l}^{l} \left| q_{lm}\left( i \right) \right|^{2} \right)^{3/2}\#\left( 17 \right) \end{aligned}$$

$$\begin{aligned} q_{lm}\left( i \right)=\frac{1}{N}\sum_{j\in\boldsymbol{N}_{b}\left( i \right)} Y_{lm}\left( \boldsymbol{r}_{ij} \right)\#\left( 18 \right) \end{aligned}$$

where $l$ is an arbitrary positive integer denoting the degree of the harmonic function, $m$ is an integer that ranges over $-l$ to $+l$, and $Y_{lm}$ is a spherical harmonic function. We consider $l=4, 6, 8, 9, 10, 11, 13,$and $15$. The locally averaged bond-orientational order parameters $\bar{Q}^{S}$ and $\bar{W}^{S}$ are defined as

$$\begin{aligned} \bar{Q}_{l}^{S}\left( i \right)=\frac{1}{N+1}\sum_{j\in{\tilde{\boldsymbol{N}}}_{b}\left( i \right)} Q_{l}^{S}\left( j \right)\#\left( 19 \right) \end{aligned}$$

$$\begin{aligned} \bar{W}_{l}^{S}\left( i \right)=\frac{1}{N+1}\sum_{j\in{\tilde{\boldsymbol{N}}}_{b}\left( i \right)} W_{l}^{S}\left( j \right)\#\left( 20 \right) \end{aligned}$$

The modified bond-orientational order parameters $\bar{Q}^{L}$ and $\bar{W}^{L}$, locally averaged over the spherical harmonic function term $q_{lm}$, are expressed as

$$\begin{aligned} Q_{l}^{L}\left( i \right)=\sqrt{\frac{4\pi}{2l+1}\sum_{m=-l}^{l} \left| \bar{q}_{lm}\left( i \right) \right|^{2}}\#\left( 21 \right) \end{aligned}$$

$$\begin{aligned} W_{l}^{L}\left( i \right)=\sum_{m_{1}+m_{2}+m_{3}=0} \left( \begin{matrix} l & l & l \\ m_{1} & m_{2} & m_{3} \end{matrix} \right)\bar{q}_{lm_{1}}\left( i \right)\bar{q}_{lm_{2}}\left( i \right)\bar{q}_{lm_{3}}\left( i \right)/\left( \sum_{m=-l}^{l} \left| \bar{q}_{lm}\left( i \right) \right|^{2} \right)^{3/2}\#\left( 22 \right) \end{aligned}\begin{aligned} \# \end{aligned}$$

$$\begin{aligned} \bar{q}_{lm}\left( i \right)=\frac{1}{N+1}\sum_{j\in{\tilde{\boldsymbol{N}}}_{b}\left( i \right)} q_{lm}\left( j \right)\#\left( 23 \right) \end{aligned}$$

The locally averaged modified bond-orientational order parameters $\bar{Q}^{L}$ and $\bar{W}^{L}$ are defined as

$$\begin{aligned} \bar{Q}_{l}^{L}\left( i \right)=\frac{1}{N+1}\sum_{j\in{\tilde{\boldsymbol{N}}}_{b}\left( i \right)} Q_{l}^{L}\left( j \right)\#\left( 24 \right) \end{aligned}$$

$$\begin{aligned} \bar{W}_{l}^{L}\left( i \right)=\frac{1}{N+1}\sum_{j\in{\tilde{\boldsymbol{N}}}_{b}\left( i \right)} W_{l}^{L}\left( j \right)\#\left( 25 \right) \end{aligned}$$

The alternative bond-orientational order parameters $LQ$ and $LW$, for which $q_{lm}$ was normalized, are expressed as

$$\begin{aligned} {LQ}_{l}\left( i \right)=\frac{1}{N}\sum_{j\in\boldsymbol{N}_{b}\left( i \right)} \frac{\sum_{m=-l}^{l} q_{lm}\left( i \right)q_{lm}^{*}\left( j \right)}{\left| \sum_{m=-l}^{l} q_{lm}\left( i \right)q_{lm}^{*}\left( j \right) \right|\left| \sum_{m=-l}^{l} q_{lm}\left( j \right)q_{lm}^{*}\left( i \right) \right|}\#\left( 26 \right) \end{aligned}$$

$$\begin{aligned} {LW}_{l}\left( i \right)=\sum_{m_{1}+m_{2}+m_{3}=0} \left( \begin{matrix} l & l & l \\ m_{1} & m_{2} & m_{3} \end{matrix} \right){lq}_{lm_{1}}\left( i \right){lq}_{lm_{2}}\left( i \right){lq}_{lm_{3}}\left( i \right)/\left( \sum_{m=-l}^{l} \left| {lq}_{lm}\left( i \right) \right|^{2} \right)^{3/2}\#\left( 27 \right) \end{aligned}$$

$$\begin{aligned} {lq}_{lm}\left( i \right)=\frac{1}{N}\sum_{j\in\boldsymbol{N}_{b}\left( i \right)} \frac{q_{lm}\left( i \right)q_{lm}^{*}\left( j \right)}{\left| q_{lm}\left( i \right) \right|\left| q_{lm}\left( j \right) \right|}\#\left( 28 \right) \end{aligned}$$

The locally averaged alternative bond-orientational order parameters $\bar{LQ}$ and $\bar{LW}$ are defined as

$$\begin{aligned} \bar{LQ}_{l}\left( i \right)=\frac{1}{N+1}\sum_{j\in{\tilde{\boldsymbol{N}}}_{b}\left( i \right)} LQ_{l}\left( j \right)\#\left( 29 \right) \end{aligned}$$

$$\begin{aligned} \bar{LW}_{l}\left( i \right)=\frac{1}{N+1}\sum_{j\in{\tilde{\boldsymbol{N}}}_{b}\left( i \right)} LW_{l}\left( j \right)\#\left( 30 \right) \end{aligned}$$

The modified alternative bond-orientational order parameters $LQ^{T}$ and $LW^{T}$, locally averaged over the ${lq}_{lm}$ of $LQ$ and $LW$, are expressed as

$$\begin{aligned} {LQ}_{l}^{T}\left( i \right)=\frac{1}{N}\sum_{j\in\boldsymbol{N}_{b}\left( i \right)} \frac{\sum_{m=-l}^{l} \bar{q}_{lm}\left( i \right)\bar{q}_{lm}^{*}\left( j \right)}{\left| \sum_{m=-l}^{l} \bar{q}_{lm}\left( i \right)\bar{q}_{lm}^{*}\left( j \right) \right|\left| \sum_{m=-l}^{l} \bar{q}_{lm}\left( j \right)\bar{q}_{lm}^{*}\left( i \right) \right|}\#\left( 31 \right) \end{aligned}$$

$$\begin{aligned} {LW}_{l}^{T}\left( i \right)=\sum_{m_{1}+m_{2}+m_{3}=0} \left( \begin{matrix} l & l & l \\ m_{1} & m_{2} & m_{3} \end{matrix} \right)\bar{lq}_{lm_{1}}\left( i \right)\bar{lq}_{lm_{2}}\left( i \right)\bar{lq}_{lm_{3}}\left( i \right)/\left( \sum_{m=-l}^{l} \left| \bar{lq}_{lm}\left( i \right) \right|^{2} \right)^{3/2}\#\left( 32 \right) \end{aligned}$$

$$\begin{aligned} \bar{lq}_{lm}\left( i \right)=\frac{1}{N+1}\sum_{j\in{\tilde{\boldsymbol{N}}}_{b}\left( i \right)} {lq}_{lm}\left( j \right)\#\left( 33 \right) \end{aligned}$$

The locally averaged alternative bond-orientational order parameters $\bar{LQ}^{T}$ and $\bar{LW}^{T}$ are defined as

$$\begin{aligned} \bar{LQ}_{l}^{T}\left( i \right)=\frac{1}{N+1}\sum_{j\in{\tilde{\boldsymbol{N}}}_{b}\left( i \right)} LQ_{l}^{T}\left( j \right)\#\left( 34 \right) \end{aligned}$$

$$\begin{aligned} \bar{LW}_{l}^{T}\left( i \right)=\frac{1}{N+1}\sum_{j\in{\tilde{\boldsymbol{N}}}_{b}\left( i \right)} LW_{l}^{T}\left( j \right)\#\left( 35 \right) \end{aligned}$$

[1] Takahashi, K. Z. Molecular cluster analysis using local order parameters selected by machine learning. Phys. Chem. Chem. Phys. 25, 658–672, (2023).
